# Seasonal shifts in water-source partitioning and fine-root overlap in a *Populus simonii*–maize shelterbelt system

**DOI:** 10.3389/fpls.2026.1968926

**Published:** 2026-09-23

**Authors:** Jianuo Zhang, Zerui Tan, Han Yan, Fengbin Sun

**Affiliations:** 1 North China University of Water Resources and Electric Power, Zhengzhou, China; 2 Co‐Innovation Center for Sustainable Forestry in Southern China, Nanjing Forestry University, Nanjing, China; 3 Key Laboratory of Soil and Water Conservation and Desertification Combating of Ministry of Education, Beijing Forestry University, Beijing, China; 4 Weather Modification Centre, China Meteorological Administration, Beijing, China

**Keywords:** dry-wet seasonal comparison, farmland shelterbelt, root overlap, stable water isotopes, water-source niche

## Abstract

**Introduction:**

Farmland shelterbelts are critical for agricultural production in arid and semiarid regions, but they may compete with crops for limited soil water. This study aimed to quantify seasonal water-source use, soil moisture dynamics, and fine-root distribution in a *Populus simonii*–maize shelterbelt system.

**Methods:**

The study was conducted in Zhangbei County, northwestern Hebei Province, China. Three land-use conditions were compared: a pure *P. simonii* shelterbelt (P), a *P. simonii*–maize intercropping system (PC), and a maize monoculture reference (C). Synchronous sampling was conducted in May, July, August, and October 2023. Stable hydrogen and oxygen isotope ratios of soil water, groundwater, and plant xylem water were measured. The MixSIAR Bayesian mixing model was used to estimate water-source contributions. Fine-root length density was measured to 160 cm depth and at four horizontal distances from tree trunks. Schoener’s proportional similarity index, Pianka’s niche-overlap index, and Levins’ niche-breadth index were calculated.

**Results:**

*P. simonii* relied strongly on deep soil water and a groundwater-like endmember, with a combined contribution exceeding 55% during the dry season. Maize relied more on shallow soil water, with >60% of its water uptake from the 0–40 cm layer during the rainy season; during the dry season, its estimated contribution from 40–100 cm increased. Water-source overlap was higher in the dry season than in the rainy season. The strongest fine-root co-occurrence occurred in the 40–80 cm soil layer within 0–100 cm of the tree trunk.

**Discussion:**

*P. simonii* exhibited a broader and more stable water-source niche than maize. Maize showed a larger seasonal shift in water uptake, especially under intercropping, suggesting an asymmetric water-use response. These results identify the specific soil layers and periods where tree–crop water interactions are likely to occur. However, because the study was conducted in a single year and at one site, the findings should be treated as testable hypotheses for future multi-year, replicated irrigation and spacing experiments.

## 1 Introduction

Farmland shelterbelts serve as a critical barrier safeguarding agricultural production in arid and semiarid regions. By reducing wind speed, stabilizing soil structure, and regulating farmland microclimate, they can effectively mitigate the adverse impacts of wind erosion on crop growth (Aili et al., 2025; He et al., 2025). However, in water-scarce regions, shelterbelts may compete with crops for limited soil water while delivering ecological protective functions. When trees and crops take up water from overlapping soil layers in the soil profile, interspecific belowground water competition arises, which weakens the yield-increasing effect of shelterbelts and even suppresses crop growth in severe cases (Li et al., 2020; Ma et al., 2022; Swieter et al., 2022). Compared with treeless farmland, woody plants increase total ecosystem transpiration (Xiao and Huang, 2016), so irrationally configured shelterbelt–crop systems may exacerbate soil water deficits. With increasingly frequent seasonal drought events and more uneven rainfall patterns (He et al., 2023; Wu et al., 2016; Zha and Zhang, 2025), fluctuations in soil water availability continue to intensify, which may further elevate the risk of water competition between trees and crops. Accordingly, clarifying the water use relationships between shelterbelt trees and crops as well as their seasonal dynamics holds important theoretical and practical significance for the rational configuration and sustainable water management of shelterbelt systems in semiarid regions.

Stable hydrogen and oxygen isotope tracing is a robust method for quantifying plant water sources and has greatly advanced research on interspecific water relations in agroforestry systems (Chen et al., 2023). Previous studies have demonstrated that different plant species can achieve resource partitioning through vertical differentiation of water use: deep-rooted trees access deeper soil water, whereas shallow-rooted crops generally rely more on surface soil water replenished by rainfall (Liu et al., 2026a; Liu et al., 2026b; Shi et al, 2025). This “water source stratification” is regarded as a key mechanism for mitigating interspecific water competition. However, water use differentiation is not static; its specific patterns shift with season, stand structure, and soil water availability (Zhao et al., 2024). In particular, under drought stress, shallow-rooted crops may be forced to shift to deeper soil water, resulting in overlap with the water use niche of trees (Liu et al., 2026). In shelterbelt–farmland systems, existing studies have mainly focused on species configuration (Aili et al., 2024), nutrient cycling (Yang et al., 2024), and system-scale ecological benefits (Monckton and Mendham, 2022), yet two key uncertainties remain. First, relatively few studies have integrated seasonal water source estimates with horizontal distance from shelterbelts (Huo et al., 2021). Second, root distribution characteristics and isotope-inferred water use patterns are often investigated separately, which limits mechanistic explanations for the specific spatial locations and temporal dynamics of water use overlap (Schmutz and Schöb, 2023).

We therefore examined seasonal water-source use, soil moisture, and fine-root distribution in a *Populus simonii*–maize shelterbelt system. We tested three predictions: (1) *P. simonii* would rely more strongly on deep soil water, whereas maize would rely more strongly on shallow soil water; (2) maize would shift its inferred water uptake downward during dry-season, increasing water-source overlap with *P. simonii*; and (3) fine-root co-occurrence would be greatest in intermediate soil layers close to the shelterbelt. The study was designed to characterize seasonal associations among soil-water availability, water-source use, and root distributions, and to identify management hypotheses that can be tested in replicated irrigation and spacing experiments.

## 2 Materials and methods

### 2.1 Study area

The study was conducted in Zhangbei County (40°57′–41°34′ N, 114°10′–115°27′ E), northwestern Hebei Province, China. The region has a mid-temperate continental monsoon climate and a mean elevation of approximately 1,750 m. Mean annual precipitation is 392.7 mm, most of which falls from June to September, and mean annual temperature is 4.1 °C. Mean annual wind speed is 4 m s^−1^, and winds above force 7 occur on an average of 58 days per year. The dominant soil type is chestnut soil. Groundwater occurs at a depth greater than 15 m and was included as an isotopic endmember.

### 2.2 Experimental design

Three land-use conditions were compared: a pure *P. simonii* shelterbelt (P), a *P. simonii*–maize intercropping system (PC), and a maize monoculture reference (C). Twelve 20 m × 20 m plots were sampled, with four plots representing each condition. Three sampling points were randomly selected within each plot and treated as within-plot subsamples. The P and PC plots were located in contiguous shelterbelt areas, whereas the four C plots were located in farmland approximately 1.7 km away and outside the apparent influence of the shelterbelt. Although the C plots share the same soil type and regional farming practices, this spatial separation inevitably introduces local variability; we therefore define C as a “spatial monoculture reference” rather than a strict experimental control. To minimize the potential for root connectivity among blocks, a buffer interval of at least 20 m was maintained between adjacent replicate blocks within the contiguous shelterbelt and intercropping areas. The plot was treated as the primary sampling unit, and the three sampling points were nested within plots.

The selected *P. simonii* stand was approximately 15 years old, with a mean tree height of 12.0 m and a mean diameter at breast height of 17.2 cm. *P. simonii* was planted at 4 m × 5 m spacing, and maize was planted at 0.5 m row spacing and 0.6 m within-row spacing; in the intercropping system, the maize rows nearest to the shelterbelt were approximately 1.5 m from the tree row, following the standard local configuration. Field management followed local conventional practices.

*P. simonii* and maize shared a growing season from April to November. Synchronous sampling was conducted in May, July, August, and October 2023. Based on the regional precipitation regime, May and October were classified as dry-season, and July and August as rainy-season. May and October were treated as two separate dry-season, rather than as successive points along a continuous drying trajectory. It should be emphasized that all sampling was conducted within a single growing season. Thus, the “dry-season” and “rainy-season” designations apply specifically to the precipitation pattern of the study year; distinguishing between consistent seasonal patterns and interannual climatic anomalies would require replicated observations across multiple years.

### 2.3 Stable isotope sample collection

#### 2.3.1 Plant xylem sample collection

Sampling was conducted during 17 May, 22 July, 18 August, and 15 October 2023 on clear days. To minimize the influence of recent precipitation on isotopic signatures, all sampling was conducted on clear days following at least three consecutive rain-free days. At each sampling date, three healthy plants of similar size were selected at the three sampling points within each of the four plots. This yielded 12 plant-level subsamples per condition at each date. For *P. simonii*, one-year-old lignified branches were collected from the sunlit upper canopy; for maize, 3–5 cm sections of lignified basal stem were collected. Both sample types are woody, non-photosynthetic, and non-transpiring tissues, avoiding evaporative enrichment from leaf transpiration. Because root water uptake and xylem transport involve no hydrogen or oxygen isotope fractionation, xylem water in both reflects the integrated mixed signal of root-absorbed water, satisfying the assumptions of the isotope mixing model (MixSIAR). Green tissues and phloem were removed, and samples were sealed immediately in 20 mL glass vials with Parafilm^®^, transported in a cooled container, and stored at 4 °C until xylem-water extraction.

#### 2.3.2 Soil sample collection

Soil and plant sampling were conducted synchronously, with three soil profile samples collected near each target plant using a soil auger. The cores represented technical subsamples at each sampling point and were summarized within plots for statistical analysis. Each core was divided into seven depth intervals: 0–20, 20–40, 40–60, 60–80, 80–100, 100–120, and 120–160 cm. One subsample was used to determine gravimetric soil-water content by oven drying at 105 °C. The remaining soil was sealed in centrifuge tubes with Parafilm^®^ and frozen until stable-isotope analysis.

#### 2.3.3 Groundwater sample collection

Groundwater was sampled biweekly from a nearby domestic well. The well taps the same unconfined aquifer that underlies the study plots. Because the water table remained >15 m below the surface throughout the study period, while the maximum root sampling depth was 1.6 m, we treated groundwater as a groundwater-like isotopic endmember in the mixing model, rather than as a direct root-accessible water source.

### 2.4 Determination of stable hydrogen and oxygen isotope ratios

The calculations used in this study are described by Equations 1–7. Water was extracted from plant and soil samples at the Chinese Academy of Forestry using a LI-2100 automated vacuum extraction system. The extraction temperature was 180 °C and the condensation temperature was −80 °C. Stable hydrogen and oxygen isotope ratios were measured using a Thermo Scientific™ EA IsoLink™ isotope-ratio mass spectrometer. Isotope ratios are reported in per mil (‰) relative to Vienna Standard Mean Ocean Water. Analytical precision was ±0.3‰ for δ²H and ±0.1‰ for δ¹^8^O. For each isotope separately, isotope values were calculated using Equation 1 as follows:

(1) $$\delta^{2}H/\delta^{18}O=\left(\frac{R_{sample}}{R_{\text{standard}}}-1\right)\times 1000‰$$

where R_sample_ and R_standard_ are the isotope ratios of the sample and Vienna Standard Mean Ocean Water, respectively.

### 2.5 Quantitative partitioning of plant water sources based on stable isotopes

The MixSIAR Bayesian mixing model was used to estimate the proportional contributions of candidate water sources to plant xylem water using δ²H and δ¹^8^O. Isotopic fractionation during root water uptake was assumed to be zero.

The model was fitted using Markov chain Monte Carlo sampling with three independent chains, 200,000 iterations, and a burn-in of 50,000 iterations. Convergence was evaluated using the Gelman–Rubin and Geweke diagnostics, which indicated adequate chain convergence. Model adequacy was confirmed through residual analysis, posterior predictive checks, and goodness-of-fit statistics, all of which indicated a satisfactory fit. Source contributions were summarized from the posterior distributions rather than from posterior means alone.

The seven sampled soil layers were aggregated into three soil-water endmembers (0–40, 40–100, and 100–160 cm) based on soil hydrological properties and vertical isotope patterns; groundwater was included as a fourth, groundwater-like endmember. This three-layer division reflected both vertical isotope stratification and hydrological functionality: the 0–40 cm layer was most enriched in heavy isotopes and most dynamic in response to rainfall; the 40–100 cm layer served as a transitional buffer; and the 100–160 cm layer exhibited stable, depleted isotope signatures approaching the groundwater-like endmember. This aggregation balanced ecologically meaningful source separation with sufficient sample sizes for robust MixSIAR modeling. Because the 100–160 cm soil-water and groundwater-like endmembers had similar isotope signatures, their separate contributions were not treated as independently identifiable. Posterior draws for these two endmembers were therefore summed, and the pooled deep/groundwater-like contribution was used for biological interpretation. The mass-balance equations were as follows (Equations 2, 3):

(2) $$\delta X=c_{1}\delta X_{1}+c_{2}\delta X_{2}+c_{3}\delta X_{3}+c_{4}\delta X_{4}$$

(3) $$c_{1}+c_{2}+c_{3}+c_{4}=1$$

where X is the δ²H or δ¹^8^O value of plant xylem water (‰); X_1_, X_2_, X_3_, and X_4_ are the corresponding isotope values of water from 0–40, 40–100, and 100–160 cm soil and the groundwater-like endmember, respectively; and c_1_, c_2_, c_3_, and c_4_ are their estimated proportional contributions.

### 2.6 Measurement of fine root length density in *P. simonii*-maize shelterbelt systems

At the end of the growing season (October), roots were sampled in each of the four plots per condition using a soil auger with an internal diameter of 10 cm. Samples were divided into eight depth intervals: 0–20, 20–40, 40–60, 60–80, 80–100, 100–120, 120–140, and 140–160 cm. In the intercropping system, samples were collected at four horizontal distances from the tree trunk (0–50, 50–100, 100–150, and 150–200 cm), with three cores per plot for each depth-by-distance combination. Horizontal distance intervals (0-50 cm, 50-100 cm, 100-150 cm, 150-200 cm) from the poplar trunk were defined based on the preliminary survey results of horizontal fine-root distribution of *Populus simonii* shelterbelts in the study area. This segmentation helps capture the gradient variation from tree-proximal zones with strong poplar root influence to tree-distal maize zones with weak root interference. The three cores were treated as within-plot subsamples. Roots were washed and assigned to *P. simonii* or maize using color, epidermal texture, and toughness. Fine roots (<2 mm diameter) were quantified with a WinRHIZO-EC root analysis system. Root length density (ρL, cm cm^−3^) using Equation 4 as follows:

(4) $$\rho L=\frac{L}{V}$$

where L and V represent the total root length (cm) and the soil core volume (cm³), respectively.

### 2.7 Quantification of water-source and fine-root niche metrics

Water-source overlap between *P. simonii* and maize was quantified using Schoener’s proportional similarity index (PS; Equation 5). Fine-root spatial overlap was quantified using Pianka’s symmetric niche-overlap index, and water-source niche breadth was quantified using the unstandardized Levins index. Higher overlap values indicate greater similarity in resource use or root distribution; they should not, by themselves, be interpreted as direct measurements of competitive effects.

(5) $$PS=1-0.5\sum_{i=1}^{n}|P_{1i}-P_{2i}|$$

where P_1__*i*_ and P_2__*i*_ are the proportional contributions of water source *i* to the two species, respectively, and *n* is the number of water-source endmembers.

Pianka’s index was used to quantify similarity in the spatial distribution of fine roots between species (Equation 6):

(6) $$Q_{ik}=\frac{\sum_{j-1}^{r}P_{ij}P_{kj}}{\sqrt{\sum_{j-1}^{r}P_{ij}^{2}\sum_{j-1}^{r}P_{kj}^{2}}}$$

where Q_ik_ is the fine-root niche overlap between species *i* and *k*; P_*ij*_ and P_*kj*_ are the proportional root length densities of species *i* and *k* in soil layer *j*, respectively; and *r* is the number of soil layers.

The unstandardized Levins niche-breadth index was used to characterize the breadth of water-source use (Equation 7):

(7) $$B_{i}=1/\sum_{j=1}^{r}Pij^{2}$$

where B_i_ is the water-source niche breadth of species i, P_ij_ is the proportional contribution of water source j to species i, and r is the number of water-source endmembers.

### 2.8 Statistical analysis

Statistical analyses were performed in SPSS 16.0 (SPSS Inc., Chicago, IL, USA). Because all sampling blocks within the *P. simonii* shelterbelt and intercropping treatments were located in the same contiguous stand and shared regional hydrological conditions, they do not qualify as fully independent fixed replicates at the landscape scale. Therefore, in the statistical models, the four blocks per condition were treated as random-effect factors to account for within-stand spatial heterogeneity, whereas planting condition, sampling period, soil depth, and their interactions were treated as fixed effects. Soil-water content and soil-water isotope composition were analyzed using repeated-measures mixed models with planting condition, sampling period, soil depth, and their interactions as fixed effects and plot as a random effect. Root length density was analyzed with planting condition or species, soil depth, horizontal distance, and their relevant interactions as fixed effects and plot as a random effect. Plot-level niche indices were analyzed using the corresponding condition and sampling-period effects. Tukey adjustment was used for *post hoc* pairwise comparisons. Residual normality and homogeneity of variance were assessed before inference, and P< 0.05 was considered statistically significant. MixSIAR source contributions were summarized as posterior estimates with 95% credible intervals; contrasts among plant groups were interpreted from posterior distributions rather than by applying ANOVA to posterior means.

## 3 Results

### 3.1 Soil moisture dynamics across dry and wet seasons

Soil-water content generally increased with depth under all planting conditions (**Figure 1**). The shallow layer (0–60 cm) varied more strongly among sampling periods than the 100–160 cm layer. During the dry-season sampling periods, profile water content ranged from 10.9% to 23.3% in maize monoculture (C), 9.2% to 22.4% in the pure *P. simonii* shelterbelt (P), and 6.9% to 21.3% in the intercropping system (PC). During Rainy-season, water content increased across the profile; in monoculture maize, water content increased by 62.5% in the 0–20 cm layer but by only 5.2% in the 120–160 cm layer. Across the measured profiles, water content followed the general order C > P > PC. In the dry-season samples, mean water content in the 0–60 cm layer was 2.6 percentage points lower in PC than in P and 4.8 percentage points lower in PC than in C. The three-way interaction (land use × season × depth) was not significant (*F* = 1.23, *P* = 0.32). However, the land use × season interaction was significant (*F* = 15.6, *P*< 0.001), with the PC treatment showing a greater dry-season SWC decline than C.

**Figure 1:**
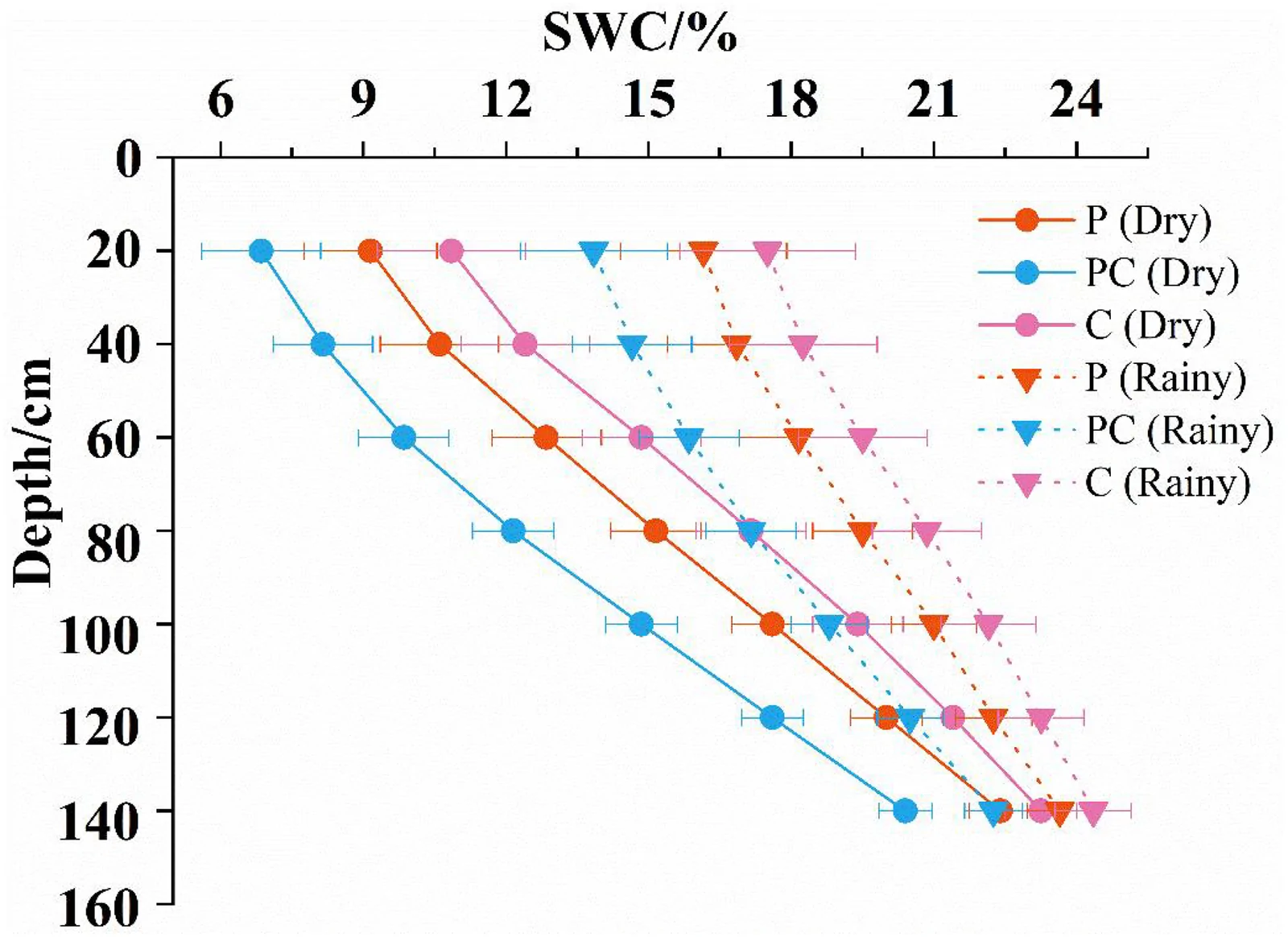
Seasonal variation in soil-water content (SWC) under the pure *Populus simonii* shelterbelt (P), *P. simonii*–maize intercropping system (PC), and maize monoculture (C). Solid lines represent samples from the dry-season (May and October), and dashed lines represent samples from the rainy-season (July and August). Values are plot-level means; error bars indicate SD among four plots (n = 4 plots per condition).

### 3.2 Root distribution patterns of *P. simonii* and maize

Fine-root distributions differed between species and planting conditions (**Figure 2**). Vertically, intercropped *P. simonii* fine-root length density increased with depth and reached 2.50 cm cm−3 at 120–160 cm within 0–50 cm of the trunk. Maize fine roots were concentrated in shallower soil, reaching 3.00 cm cm−3 in the 20–40 cm layer at 50–100 cm from the trunk, and were nearly absent below 120 cm. Horizontally, *P. simonii* fine-root length density decreased with distance from the trunk, whereas maize fine-root length density peaked at 50–100 cm. The strongest fine-root co-occurrence occurred in the 40–80 cm layers within 0–100 cm of the trunk. Relative to monoculture, intercropping was associated with a greater proportion of shallow *P. simonii* fine roots and lower overall maize fine-root length density.

**Figure 2:**
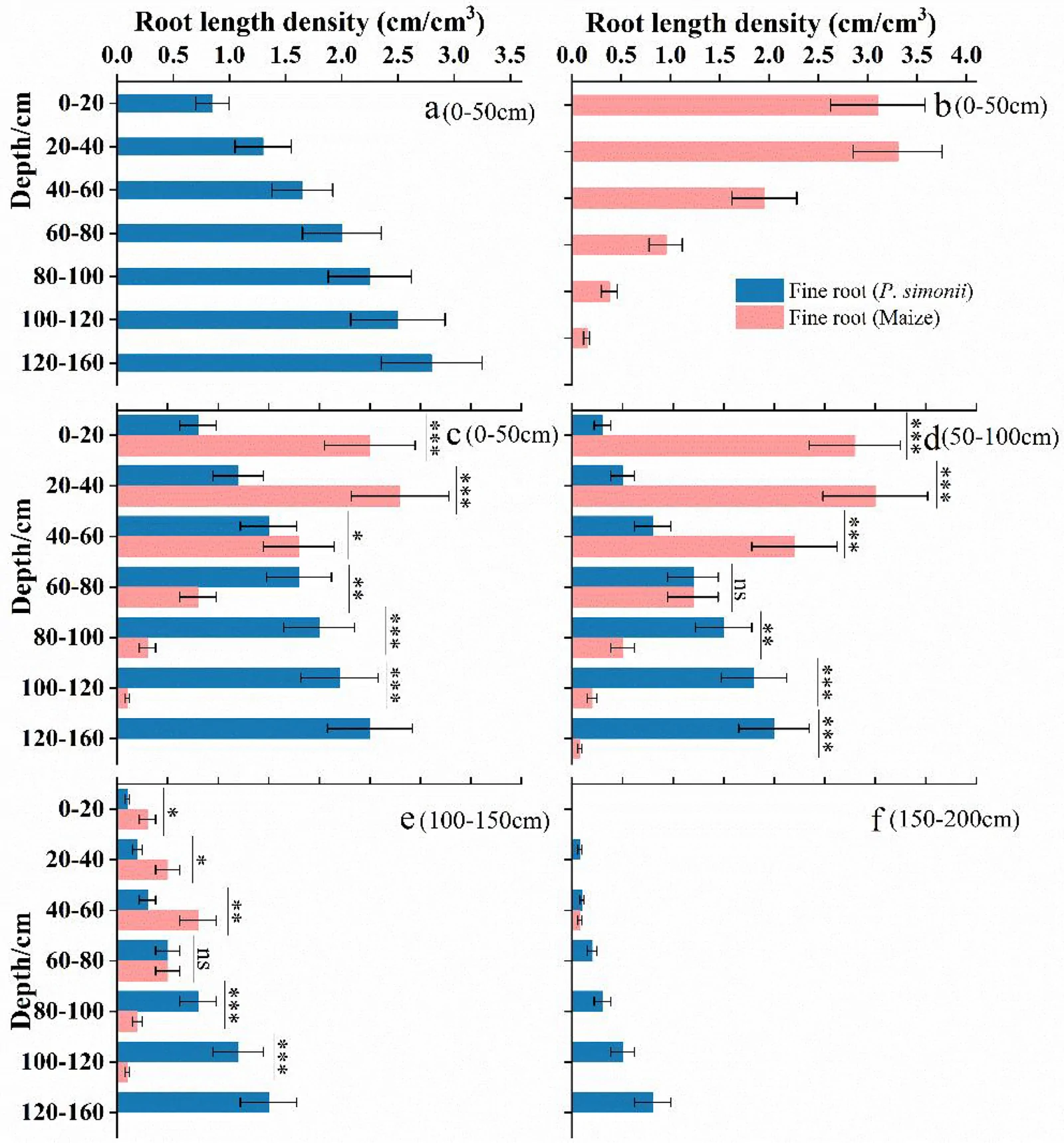
Root length density of *Populus simonii* and maize under the three planting conditions. Panels **(A, B)** show the pure shelterbelt and maize monoculture reference, respectively; panels **(C-F)** show intercropped *P. simonii* and maize across the four horizontal distance classes. Fine and coarse roots were defined as<2 mm and >2 mm in diameter, respectively. Values are plot-level means; error bars indicate SD among four plots (n = 4 plots per condition). ns = not significant; *P< 0.05; **P< 0.01; ***P< 0.001.

### 3.3 Stable isotope compositions of water pools

Soil-water δ²H and δ¹^8^O showed vertical and seasonal variation: shallow soil water was more enriched in heavy isotopes, whereas deep soil water approached the groundwater isotope signature (**Figure 3**). In dry-season samples, δ¹^8^O in the 0–20 cm layer of PC (−1.7‰) was 0.4‰ and 0.7‰ more positive than in C (−2.1‰) and P (−2.4‰), respectively. Differences in δ²H were larger, consistent with stronger evaporative enrichment in PC. During July and August, shallow-soil δ¹^8^O decreased by 2.8–3.3‰, and differences among planting conditions narrowed. Seasonal variation below 100 cm was<0.5‰. The sampled groundwater had mean δ¹^8^O and δ²H values of −10.2‰ and −85.0‰, respectively.

**Figure 3:**
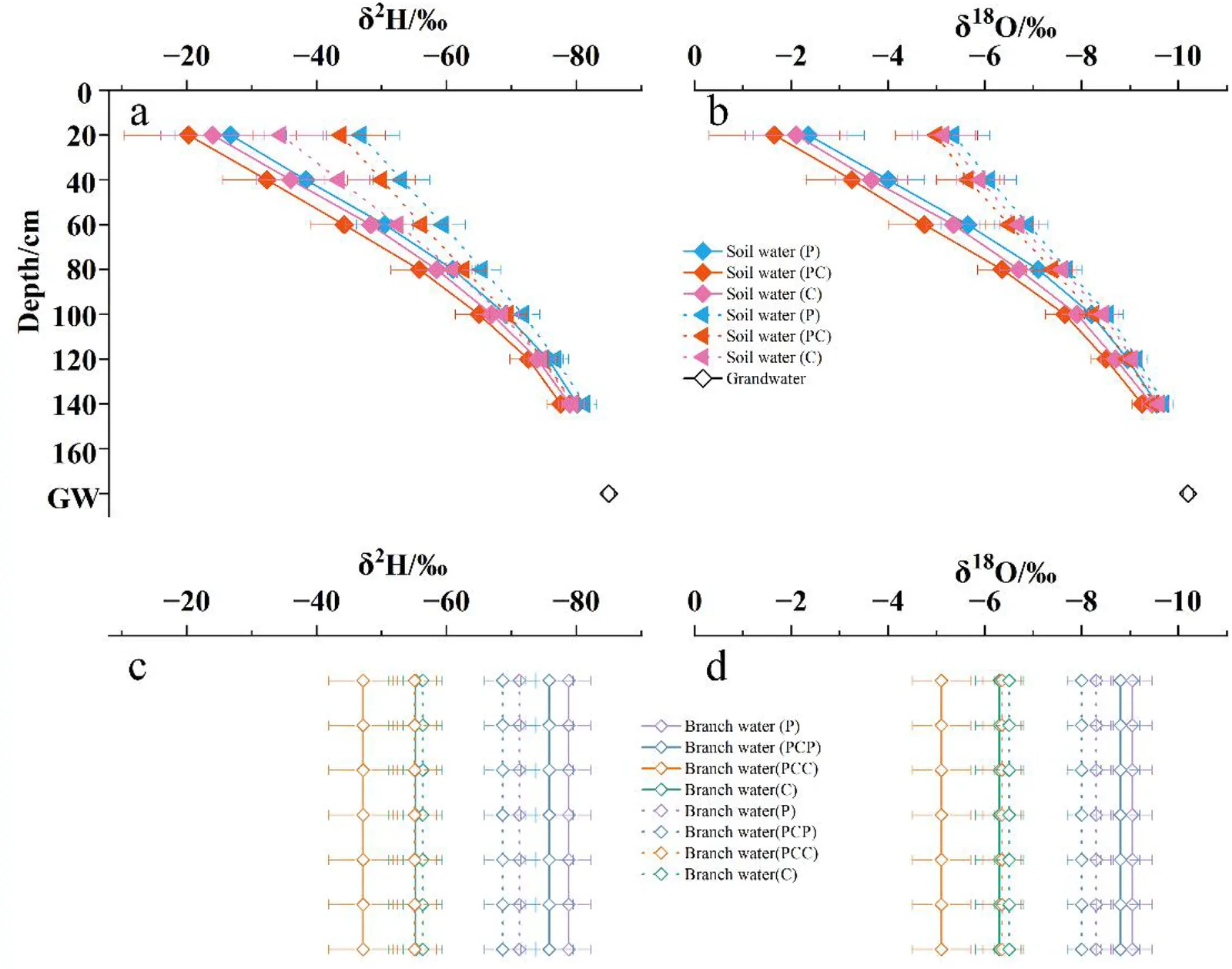
Seasonal variation in δ²H and δ¹^8^O values of soil water and xylem water under the pure *P. simonii* shelterbelt (P), *P. simonii*–maize intercropping system (PC), and maize monoculture (C). In the intercropping system, *P. simonii* and maize are denoted PCP and PCC, respectively. Panels **(a)** and **(b)** show the hydrogen and oxygen isotope values of soil water, while panels **(c)** and **(d)** show those of plant branch xylem water. Solid and dashed lines represent dry- and rainy-season samples. Values are plot-level means; error bars indicate SD among four plots (n = 4 plots per condition).

### 3.4 Plant water use sources across dry and wet seasons

MixSIAR estimates indicated different water-source patterns between *P. simonii* and maize (**Figure 4**). In the dry-season samples, the combined estimated contribution of 100–160 cm soil water and the groundwater-like endmember to *P. simonii* exceeded 55%, and the posterior estimates were similar between the pure and intercropped conditions. For maize, the estimated contribution of 0–40 cm soil water was 43.4% in monoculture and 32.9% in intercropping, whereas the 40–100 cm contribution was 49.4% in intercropping, 7.1 percentage points higher than in monoculture. During July and August, the estimated 0–40 cm contribution was 59.4% for intercropped maize and 63.9% for monoculture maize. Sources below 100 cm contributed an estimated 18.9% to intercropped maize in the dry-season samples, approximately 1.8 times the corresponding estimate for monoculture maize. Because the deep-soil and groundwater-like endmembers were isotopically similar, their pooled posterior contribution is emphasized. The interaction between season and treatment was significant for the proportional contribution of water from the middle soil layer (40–100 cm, *F* = 33.94, *P<* 0.001) and from groundwater (*F* = 9.11, *P* = 0.006), but not for the top (0–40 cm) or deep (100–160 cm) soil layers.

**Figure 4:**
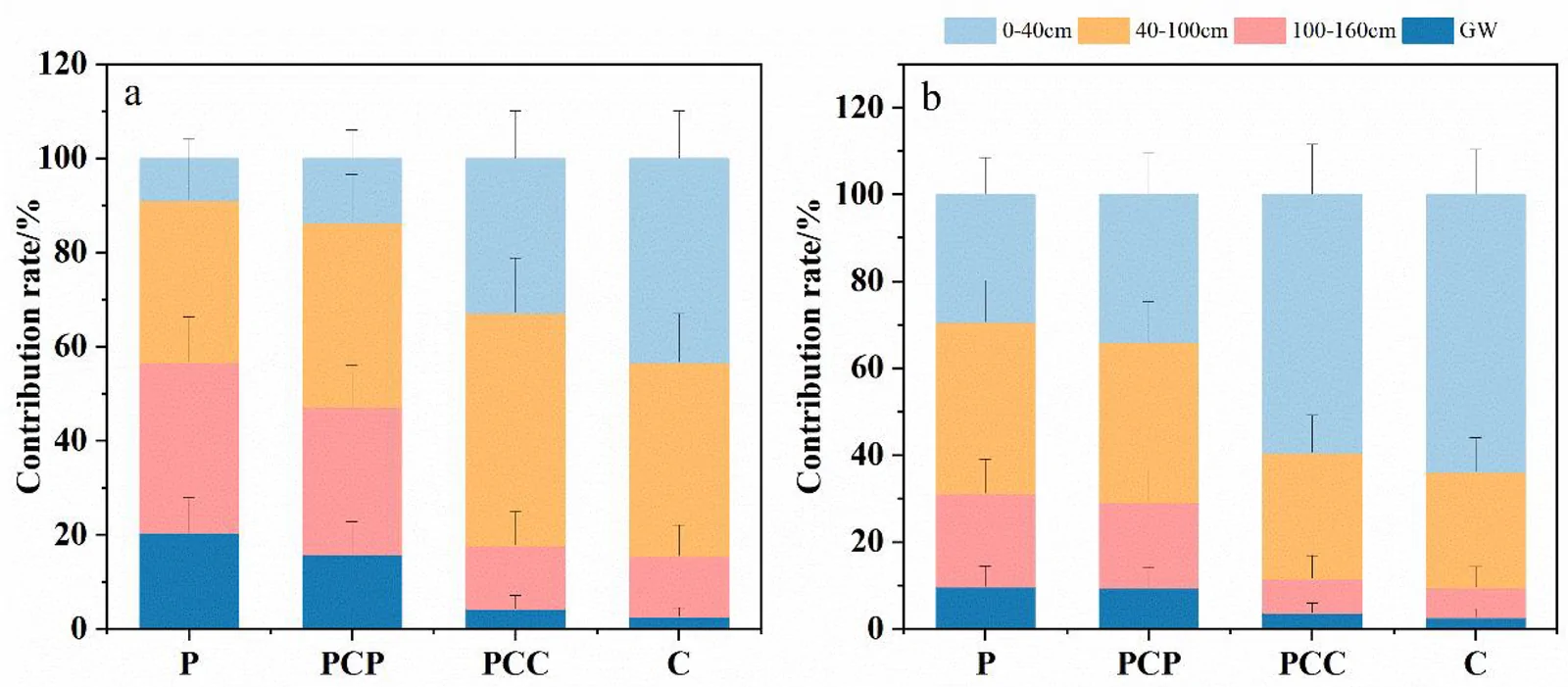
Posterior estimates of the proportional contributions of candidate water sources to *Populus simonii* and maize under different planting conditions. Panel **(a)** represents dry-season samples, and panel **(b)** represents rainy-season samples. P, pure *P. simonii* shelterbelt; PCP, *P. simonii* in the intercropping system; PCC, maize in the intercropping system; C, maize monoculture. Bars show posterior means and error bars show 95% credible intervals.

### 3.5 Seasonal variation in water-source and fine-root overlap

The niche indices of water source use and fine root distribution exhibited pronounced seasonal variation, while also reflecting significant differences in water source niche breadth between trees and crops (**Figure 5**). Schoener’s water-source overlap index was higher in the dry-season samples (0.58–0.62) than in the rainy-season samples (0.35–0.38; P< 0.05). Pianka’s fine-root overlap index was 0.48. Maize niche breadth did not differ between planting conditions during the rainy-season sampling period. During the dry-season, maize niche-breadth values varied between dates and planting conditions (2.05–2.32). Intercropped maize had a late-season value of 2.15 compared with 2.32 in monoculture (P< 0.05). *P. simonii* niche breadth ranged from 2.78 to 3.35 and was higher than that of maize (P< 0.01).

**Figure 5:**
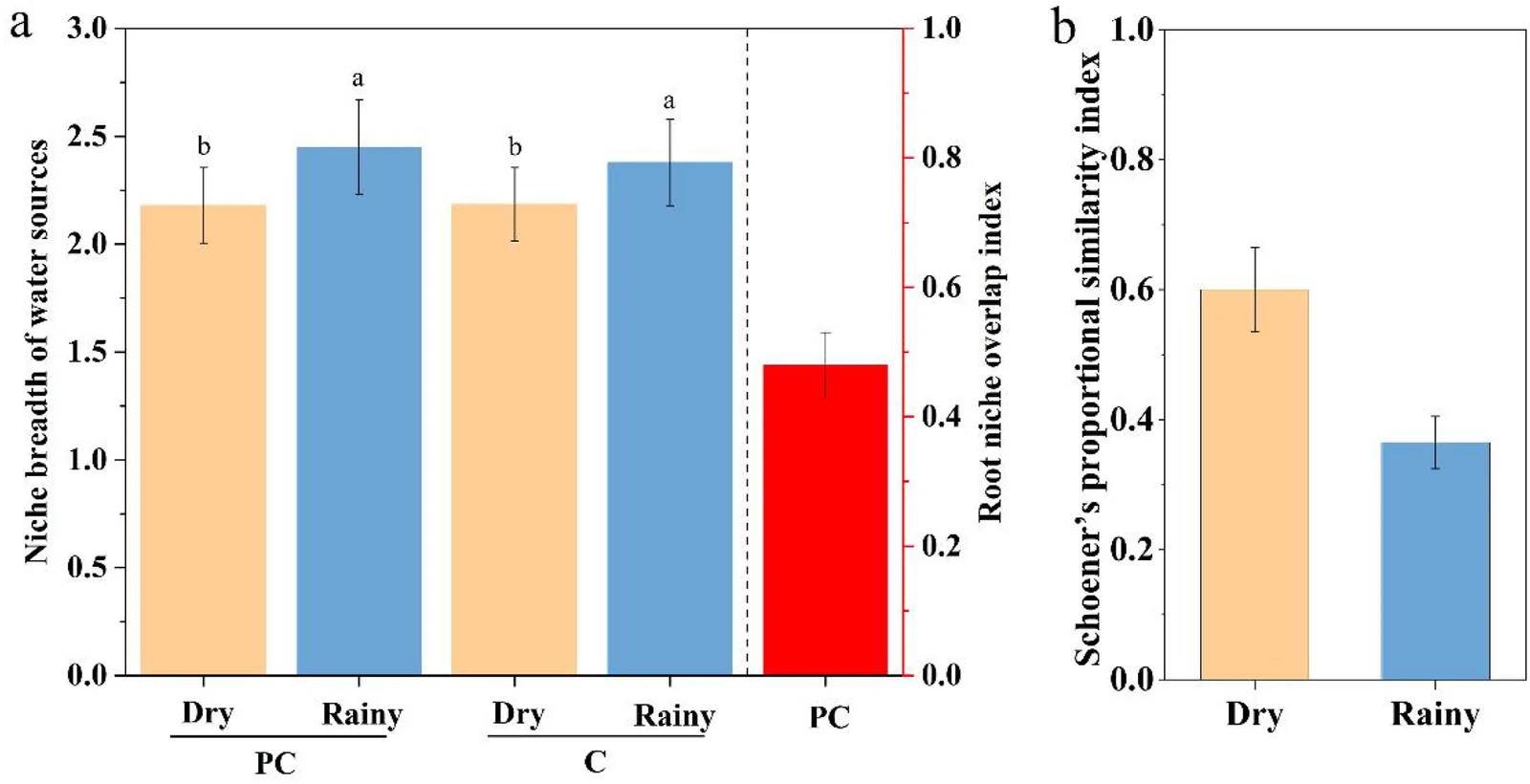
Seasonal variation in water-source niche breadth and niche overlap. Panel **(a)** shows unstandardized Levins water-source niche breadth (left y-axis) and Pianka’s fine-root niche-overlap index (right y-axis). Panel **(b)** shows Schoener’s proportional similarity index (PS) for water-source use. Bars show plot-level means ± SD (n = 4 plots per condition). Different lowercase letters indicate Tukey-adjusted pairwise differences at P< 0.05.

## 4 Discussion

### 4.1 Species-specific water-use strategies and their modulation by intercropping

Stable-isotope estimates indicated contrasting water-source patterns for shelterbelt *P. simonii* and intercropped maize. *P. simonii* showed a deep-water-use pattern: the combined estimated contribution of 100–160 cm soil water and the groundwater-like endmember exceeded 55% in the dry-season samples. The similar posterior estimates for pure and intercropped *P. simonii* suggest that its inferred water-source pattern was comparatively stable across planting conditions. This could be attributed to the fact that deep-rooted woody plants typically exhibit enhanced hydraulic buffering capacity, enabling them to draw on more stable deep water reserves and buffer against the impacts of seasonal rainfall variability (Li et al., 2025; Stocker et al., 2023; Zhao et al., 2024). However, because roots were measured only to 1.6 m and groundwater occurred below 15 m, the isotope results do not independently demonstrate direct groundwater access.

Maize showed a shallower and more seasonally variable water-source pattern. During July and August, >60% of its inferred water uptake was supplied by 0–40 cm soil water. In the dry-season samples, the estimated contribution from shallow soil declined and the contribution from 40–100 cm soil increased. This pattern is consistent with seasonal variation in crop water uptake (He et al., 2024), although the present data do not distinguish active root adjustment from changes in source-water availability.

During the dry season, intercropping induced maize to shift its water uptake strategy from surface sources toward deeper reserves. The 0–40 cm contribution was 10.5 percentage points lower in intercropped than monoculture maize, whereas the 40–100 cm contribution was 7.1 percentage points higher. This could result from active root adjustment, as the presence of tree roots induces maize to allocate more biomass to deeper soil layers—either through root plasticity or by occupying microhabitats with weaker potential competitive risk. It may also reflect passive water redistribution: evapotranspiration by poplars (transpiration plus soil evaporation) lowers shallow soil water content, thereby compelling maize to use residual deep soil water. This difference may indicate deeper uptake or water redistribution within the soil profile, consistent with context-dependent root-water uptake in agroforestry systems (Guo and Zhao, 2021); however, isotope mixing alone cannot distinguish these mechanisms. During July and August, both planting conditions returned to stronger use of shallow water, suggesting that the treatment difference was seasonal (Chen et al., 2023).

The combined contribution of 100–160 cm soil water and the groundwater-like endmember was greater for *P. simonii* than for maize. Because these two endmembers had similar isotope signatures and the root survey did not extend to the water table, they were not interpreted as independently resolved sources. The result therefore indicates use of a deep or groundwater-like isotopic source, not direct groundwater uptake.

### 4.2 Coupling of root spatial overlap and water-use overlap

Water-source overlap was higher in the dry-season samples than in the rainy-season samples, and this difference coincided with lower shallow-soil water content. The observed association is consistent with reduced water-source partitioning under seasonal water limitation (Kui et al., 2025). Previous studies have shown that the hydrological niche of plant communities is dynamic: in relatively wet years, species occupy distinct niches, resulting in hydrological niche separation; however, in dry years, all species increasingly depend on deep soil water, leading to greater niche overlap (Zhao et al., 2024). This is consistent with the patterns observed in the intercropping system in the present study. The value of approximately 10% should therefore be treated as an exploratory, site-specific observation rather than a threshold and should be tested using denser time-series data and a prespecified breakpoint model.

The spatial correspondence between fine root overlap and water source overlap provides the most direct evidence for potential water interaction zones between trees and crops. The strongest fine-root co-occurrence occurred in the 40–80 cm soil layer within 0–100 cm of the trunk (Pianka overlap = 0.48), where soil water content was also relatively low in the intercropping system. During the dry period, the water uptake ranges of both plant species contracted, and both shifted to use this soil layer, thereby forming a positive feedback loop: intensified root overlap accelerated soil water depletion, which in turn promoted further root proliferation in this layer, ultimately creating the zone of fastest soil water consumption. This spatial correspondence identifies a plausible zone of water-use interaction, but root co-occurrence alone does not demonstrate competitive suppression. Concurrent measurements of plant water status, growth, or yield would be needed to quantify the biological effect of this overlap (Guo and Zhao, 2021; Zhu et al., 2024).

Maize niche breadth varied among and planting conditions. The available observations are compatible with temporary broadening of water-source use followed by narrowing later in the season, but May and October represent separate dry-season periods interrupted by summer rainfall. A four-date, one-year data set does not establish a continuous nonlinear response to increasing drought. The expansion–compression mechanism is therefore presented as a testable interpretation rather than a demonstrated trajectory (Hoffmeister et al., 2024; Ivezic et al., 2021; Scordia et al., 2023).

### 4.3 Methodological uncertainty and implications for shelterbelt management

Several methodological limitations constrain the generalizability of our findings. Because this study lasted one year and used a single site, the observation period could not capture a complete multi-year climatic sequence, making it difficult to determine whether the observed dry-season/wet-season differences reflect intrinsic seasonal patterns or inter-annual climatic variability. The shelterbelt and maize monoculture plots were ca. 1.7 km apart and were not strictly homogeneous; differences in soil texture, baseline nutrients, slope and microtopography, and long-term management history (e.g., fertilization, tillage, irrigation) may be confounded with the treatment effect and thus bias between-treatment comparisons. To reduce this risk, the maize monoculture was used as a spatial reference rather than a strict experimental control, so between-treatment differences should be interpreted as comparative trends rather than quantified causal effects. In addition, root sampling reached only 1.6 m while the water table is deeper than 15 m; the isotopic data therefore cannot directly demonstrate that *P. simonii* accesses groundwater.

*P. simonii* exhibited a broader and more stable inferred water-source niche than maize. Since intercropping was associated with a greater shift in water use in maize than in *P. simonii*, this pattern is consistent with an asymmetric water-use response, though it does not by itself constitute direct evidence of asymmetric competitive effects on plant performance (Hoffmeister et al., 2024; Liang et al., 2022; Ma et al., 2022). May and October correspond to the seedling and maturation stages of maize, respectively, and these differing phenological stages may affect the mechanisms driving changes in water source use. However, both periods were characterized by low precipitation, limited soil water replenishment, and similar patterns of water source use, and were therefore grouped in this study as a “dry season” versus “wet season” comparison. Such pooling may obscure phenological differences, and future work should analyze early- and late-season drought separately. Fine-root length density was low at 150–200 cm from the trunk, suggesting that greater tree–crop separation may reduce root overlap in this stand (Kulmatiski, 2026). However, 150 cm should not be regarded as a critical management distance until it is validated using replicated spacing treatments and crop-yield responses. Likewise, supplemental irrigation above an inferred 10% soil-water threshold remains a management hypothesis rather than a tested recommendation. Future work should combine multi-year continuous monitoring with replicated irrigation and spacing treatments, and directly measure plant growth performance to test these management hypotheses.

## 5 Conclusion

In this one-year *Populus simonii*–maize shelterbelt study, *P. simonii* showed a broad and comparatively stable reliance on deep or groundwater-like isotopic sources, whereas maize relied more strongly on shallow soil water during rainy-season and had a greater estimated contribution from 40–100 cm sources in dry-season samples. Water-source overlap was higher in the dry-season samples, and fine-root co-occurrence was greatest in the 40–80 cm layer within 1 m of the tree trunk. The larger adjustment observed in maize is consistent with an asymmetric water-use response to seasonal soil-water limitation. These results identify the specific zones and periods in which water-use interactions between trees and crops are likely to occur.

## Data Availability

The original contributions presented in the study are included in the article/supplementary material. Further inquiries can be directed to the corresponding author.
